# Students’ understanding of teamwork and professional roles after interprofessional simulation—a qualitative analysis

**DOI:** 10.1186/s41077-017-0041-6

**Published:** 2017-04-08

**Authors:** Lena Oxelmark, Torben Nordahl Amorøe, Liisa Carlzon, Hans Rystedt

**Affiliations:** 1 Institute of Health and Care Sciences, The Sahlgrenska Academy, University of Gothenburg, PO Box 457, Gothenburg, SE 405 30 Sweden; 2 Simulation Centre West, Sahlgrenska University Hospital and University of Gothenburg, Gothenburg, Sweden; 3 Department of Education, Communication & Learning, University of Gothenburg, Gothenburg, Sweden

**Keywords:** Simulation, Interprofessional, Nursing students, Medical students, Communication

## Abstract

**Background:**

This study explores how interprofessional simulation-based education (IPSE) can contribute to a change in students’ understanding of teamwork and professional roles. A series of 1-day training sessions was arranged involving undergraduate nursing and medical students. Scenarios were designed for practicing teamwork principles and interprofessional communication skills by endorsing active participation by all team members.

**Methods:**

Four focus groups occurred 2–4 weeks after the training. Thematic analysis of the transcribed focus groups was applied, guided by questions on *what* changes in students’ understanding of teamwork and professional roles were identified and *how* such changes had been achieved.

**Results:**

The first question, aiming to identify changes in students’ understanding of teamwork, resulted in three categories: realizing and embracing teamwork fundamentals, reconsidering professional roles, and achieving increased confidence. The second question, regarding how participation in IPSE could support the transformation of students’ understanding of teamwork and of professional roles, embraced another three categories: feeling confident in the learning environment, embodying experiences, and obtaining an outside perspective.

**Conclusions:**

This study showed the potential of IPSE to transform students’ understanding of others’ professional roles and responsibilities. Students displayed extensive knowledge on fundamental teamwork principles and what these meant in the midst of participating in the scenarios. A critical prerequisite for the development of these new insights was to feel confident in the learning environment. The significance of how the environment was set up calls for further research on the design of IPSE in influencing role understanding and communicative skills in significant ways.

**Electronic supplementary material:**

The online version of this article (doi:10.1186/s41077-017-0041-6) contains supplementary material, which is available to authorized users.

## Background

There is a growing awareness that patient safety in healthcare work relies on the ability of individuals to collaborate effectively with other professionals. In response, there is an increasing quest for a definition of interprofessional competencies [1]. Among these, the understanding and appreciation of one’s own and others’ roles and responsibilities are highlighted as a core competence of interprofessional work as a prerequisite for effective communication [2]. The centrality of communication, in turn, stems from the fact that the most care-related patient injuries are caused by communication failures [3, 4]. There is increasing interest in interprofessional simulation-based education (IPSE), in which students from different professions are allowed to practice teamwork and communication skills in controlled environments [5–7]. A growing body of research suggests that IPSE is well received among students and results in positive outcomes on their attitudes and knowledge [7–10]. However, few studies have explored the possibilities of IPSE to overcome relational obstacles for teamwork in contemporary healthcare that might undermine efficient communication and teamwork, such as prevailing hierarchical structures and an unequal distribution of power relationships [11]. The need to address these aspects is underlined by research indicating that nursing and medical students’ understanding of professional roles is mainly traditional and tends to be stereotypical [12, 13]. Establishing a level of trust has been noted as necessary for promoting open communication and reflection in simulation-based training [14, 15]. Further, trust has been identified as central for effective interprofessional collaboration and the need for introducing trust-building activities through interprofessional education [15, 16].

Background for the present study was the course evaluations from 172 participants (128 nursing and 44 medical students), including 29 teams in all, showing that the interprofessional simulation sessions were highly appreciated. Almost all students stated that the training improved their communicative skills (97%) and ability to handle patients’ health problems (89%). The students rated highly what they had learned about their own and others’ professions (92 and 95%, respectively) and almost unanimously (99%) would recommend other students to take part in this training. While the results from the evaluations clearly indicated that the students found the training beneficial, it motivated a further study to gain more specific knowledge on what the students learned in terms of interprofessional teamwork and the possibilities and constraints of IPSE to contribute such outcomes.

Although interprofessional training seems to be a viable option to cause a change in student attitudes, recent studies point out communication barriers between professions as obstacles for interprofessional teamwork [17] which can also hinder the establishment of trusting relations through IPSE [18–20]. Aase et al. [18] investigated the views of students, educators, and healthcare staff on how to design interprofessional training. Although their views were mainly positive, they also expressed that unequal power relationships between nurses and physicians could hamper successful outcomes of training [18]. Another study investigating nursing and medical students’ participation in IPSE showed that hierarchies within the team as well as the lack of cross-disciplinary knowledge seemed to constrain the participants’ predisposition to speak freely and to share responsibility [19].

The present study investigates these concerns in a qualitative analysis of focus group data with undergraduate nursing and medical students after participating in IPSE. Specifically, this study aims to understand if and how IPSE can change students’ perception of each other’s professions and their understanding of teamwork principles. A further and related aim was to identify what features of the IPSE design have potential for bringing about such changes. The empirical analysis was driven by two research questions:

- What changes in students’ understanding of teamwork and professional roles can be identified through IPSE?
- How can IPSE support the transformation of students’ understanding of teamwork and of professional roles?

## Methods

### Research design and setting

The interprofessional training program involved nursing students during their last semester of a 3-year education and medical students during the final years of their five-and-a-half-year education (semesters 8–11). The objective for the 1-day training was to strengthen the competencies needed for a collaborative management of emergency situations.

A lecture preceded the training to emphasize the principles of teamwork, structured communication, and systematic management of patients in everyday but potentially life-threatening situations. To clarify and outline the focus of the training, four goals were emphasized from Crisis Resource Management (CRM) and Advanced Trauma Life Support (ATLS) principles. The explicit goals were to train (1) structured examination through ABCDE (Airway, Breathing, Circulation, Disability, Environment) and (2) structured communication through SBAR (Situation, Background, Assessment, Recommendation) and to give (3) feedback by applying closed loop communication and (4) attention to critical occurrences through “Speak up” [20–23]. Five scenarios were designed with these objectives directly relating to interprofessional collaboration (Additional file 1). To promote active participation by the students from both disciplines, all scenarios included tasks that had to be performed by each profession, both separately and in collaboration. The scenarios comprised common medical conditions (for example, confusion after postoperative bleeding, vasovagal reactions, breathing problems, and anaphylactic reactions) in various healthcare settings that the students are likely to meet in their early professional career. The training sessions utilized a three-step model: *briefing*, *scenario*, and *debriefing*.

Ten to 12 students participated in each and every day of training. The large group was divided in two smaller groups of five to six students (comprising one to two medical and four to five nursing students) that trained in parallel. Every student participated in three to five of the five scenarios during the day and observed the others. All students were introduced to the simulator room to become familiar with the environment and the simulator used (SimMan 3G, Laerdal Medical, Stavanger, Norway). The active participants were introduced to the scenario they were about to encounter through a briefing. The observers followed the scenario on a projector screen in an adjacent room with instructions to observe behaviors (favorable and non-favorable) related to the objectives. The debriefing followed the model developed by Steinwachs [24]: first, the participants were asked to express their immediate feeling (“vent”). This was followed by a discussion that was divided into three steps: (1) what went well during the scenario, (2) what could be improved, and (3) what could be learned from this scenario. The initial focus on favorable actions was intended to establish a non-threatening situation and to serve as a basis for constructive discussion on what could be improved. Participants in the scenarios and observers were encouraged to engage in discussion and feedback.

### Data collection

Focus groups were arranged to gain a deep understanding of responses to the research questions [25]. These were carried out approximately 1–2 weeks after the training since a close proximity in time to the training was preferred. To facilitate participation and avoid interfering with the usual study programs, the students attending the last two training days at the end of two subsequent semesters were invited to participate, which included 48 students. Twenty-three of these students volunteered. Four focus groups were conducted, two with nursing students (eight and six participants) and two with medical students (four and five students). The rationale for choosing uniprofessional groups was that students were anticipated to express themselves more freely with peers within their own discipline.

A guide for the focus groups was developed (see Additional file 2). Each focus group was led by one or two members of the research team (LC, LO, HR) and lasted for approximately 60 min. All focus groups were audio and video recorded and transcribed verbatim. The video recordings were stored on a server in the university and were revisited in cases of uncertainty on what was said and by whom in the interviews.

### Data analysis

Inductive thematic analysis was performed [26] with inspiration from Elo and Kyngäs [27]. The software NVivo [28] was used to organize and structure the data. The transcriptions were read by all authors several times in order to become familiar with the data and gain a sense of the whole. The data corpus was openly coded by extracting meaning units corresponding to each of the research questions. Notes and headings were written in the text while reading, and the text was read through again. This way, the headings portrayed all aspects of the content and developed the codes. The initial coding was conducted by two of the researchers (HR and LO) collaborating. The codes were grouped into potential themes to capture and describe recurrent phenomena. The themes were reviewed in relation to the coded extracts and the whole dataset to generate a thematic overview of the analysis. During the analysis, the themes were refined in an iterative process until definitions and names for each theme were given. The themes formed the basis for the presentation of the results together with a selection of illustrative quotations, relating back to the analysis and research questions [26, 27]. To strengthen credibility, the analysis was conducted independently by all authors and was then discussed and modified by all authors until a consensus and an agreement were reached [29].

## Results

The results are organized in accordance with the two research questions. The first section presents significant changes in students’ understanding of teamwork and professional roles; the second section presents the conditions that were critical to enabling such transformations of understandings.

### Students’ understanding of teamwork and professional roles

Three themes emerged from the analysis: (1) realizing and embracing teamwork fundamentals, (2) reconsidering professional roles, and (3) achieving increased confidence.

### Realizing and embracing teamwork fundamentals

A recurrent topic was the necessity of creating and maintaining a shared view of the work process. The students expressed how the leader speaking out loud enabled them to prepare for the next step in the work process and invited all team members to suggest new ideas in a joint problem-solving process. Another way of maintaining a shared view was to ask for clarifications. As one nursing student said:

If you receive an instruction, I can say that I’m not really sure how to proceed, it is really okay to ask. (Focus group 4)

Further, the students emphasized that continuous feedback enabled the leader of the team (most often in the role of a physician) to maintain an overview, which in turn, allowed other team members to focus on their individual tasks. One nursing student said:

By giving feedback continuously, the physician gets the whole picture, while I, if I have to set up a drip, I can just focus on the drip for a while. Because then, I can let the rest of the situation go for awhile, while the physician has taken a step back and has an overview all the time. (Focus group 4)

Closely connected to the need to maintain an overview was the significance of leadership. Although the implication of keeping an open atmosphere and being reluctant to each others’ meanings, the students found that shared responsibility for the formation of leadership in the team was important and that the teamwork would be impaired and the structure of the team’s efforts ruined without clear leadership. This was considered to be especially important in acute situations, and if someone does not take on this responsibility, it is important for oneself to take the leadership.

The medical students expressed that a sense of trust in the nurses’ competence was necessary for maintaining an overview. The fact that nursing students had the necessary knowledge to carry out specific tasks and that the medical students themselves did not, enabled them to maintain overall responsibility for the situation. One medical student stated:

So if you just took it easy, then you realized that you could just stand here and look, and get the overview. (Focus group 1)

Similarly, the nursing students found that the accomplishment of discrete tasks connected to their role presumed trust in the leader.

### Reconsidering professional roles

The medical and nursing students expressed that they had a poor understanding of the others’ professional roles before the simulation training. One medical student recognized a lack of basic knowledge about the nursing profession:

… it’s rather embarrassing how little knowledge we have about the others’ role. (Focus group 1)

In general, the students expressed in various ways how they got a sense of what was at stake for the other profession and how they recognized their own professional responsibilities by training together. As one nursing student stated:

This became a bit more obvious in a way when collaborating with the physicians, that we have other perspectives and that our profession has other responsibilities, so that became obvious as well. (Focus group 3)

As well as knowledge about each others’ professional roles, the students also expressed new insight into their own weaknesses and strengths in working with other professions, something that highlighted the need for continuous adaptation to each other in the team. One medical student commented:

Above all, I think I learnt really, really much about myself, about strengths and weaknesses… and above all how important it is to work with another professional group for the first time, because it is really tight and you have to adjust your work in these situations. (Focus group 4)

Another crucial experience was the importance of feeling free to cross each others’ professional boundaries and to remind each other of important measures that otherwise might be forgotten (with potentially serious consequences for real patients) and that they were allowed to do so*.* As one nursing student reported:

You [referring to another nursing student] suggested a drip and when we were discussing it afterwards, although it was to stepping into the doctor's territory, the doctor thought it was really positive that you came up with a suggestion. (Focus group 4)

The students expressed the importance of such transgressions in general, and the nursing students voiced the importance for the medical students to feel supported by the rest of the team in the position as a team leader.

### Achieving increased confidence

Both nursing and medical students expressed that the IPSE increased their self-confidence. Performing as the leader for the team and knowing how to proceed conveyed the feeling that they could manage acute situations in real life. One medical student expressed that having the knowledge to manage the case put him in the position of being the leader, which in turn, led to a feeling of increased role confidence*.*

…but now I was a natural leader here, and I knew what to do, and I knew the next step. (Focus group 1)

Similarly, the nursing students found that encountering acute situations gave themselves an increased sense of confidence:

You become more secure in acute situations the more you are exposed to them, so it is really nice to have opportunities to practice. (Focus group 3)

One nursing student emphasized a development over the five scenarios during the 1-day training:

I felt strengthened when exposed to situations and experienced how I reacted to these, and I felt that I developed, just over that one day I developed a lot from the first to the last case, a kind of sense of confidence in this. (Focus group 3)

Another medical student expressed encouragement when the nursing students were pleased with his efforts:

It was really nice for me when the others told me that they felt really safe when I came in [the room]. (Focus group 2)

In parallel, nursing students, in various ways, expressed increased confidence that their competence was fundamental to successful teamwork.

### Critical conditions for the transformation of the students’ understanding

Three conditions were identified as crucial for facilitating the change of the students’ understandings: (1) feeling confident in the learning environment, (2) embodying experiences, and (3) obtaining an outside perspective.

### Feeling confident in the learning environment

The students experienced the atmosphere during the simulation training to be kind, permissive, and non-judgmental. That all participants were students created a sense of equality and contributed to a positive and easy going ambiance. This made it easier to speak up, have the courage to express feelings of doubts, and be more outspoken.

We were allowed to fail and kind of encouraged to make mistakes. There was this nice atmosphere, accepting, otherwise I think I would have felt blocked. (Focus group 4)

Important aspects that promoted a safe learning environment included working with a manikin, having clear learning goals given by the instructors, and encouraging the students to learn from their errors during simulation*.* It was also important that the team training consisted of several scenarios so that the students experienced skill improvement each time they acted in or watched a new scenario. The repeated training implied that they could foresee how the scenario would unfold. One nursing student said:

Now I feel that I understand, that I actually know what’s going on… [in critical situations]. (Focus group 3)

### Embodying experiences

The participants found that learning in the simulation environment was more real than theoretical lectures. Hands-on learning was appreciated and a complement to knowledge achieved from literature. The students expressed a sense of embodying the knowledge, that this was not merely intellectual. They described real emotional strain and feelings of stress and nervousness before the scenarios started. The stress was understood as positive for enabling learning and was described as different from the stress when taking an exam. It was good to feel nervous; the level of nervousness was not experienced as any less in the simulated environment compared with real-life situations. One of the nursing students expressed:

You kind of have the situation within your body, the patient is right there in front of you, the physician arrives and wants a handover immediately, and I just have to deliver it there and then. (Focus group 4)

The sense of embodiment and presence was associated with time aspects and logistics within the team. Dealing with parallel tasks was stressful but appreciated and perceived as central to their learning. The scenarios took realistic time; every detail around the patient was performed as if in a real clinical situation. They were obliged to perform everything, take every action step by step, and communicate as a team: nothing could be hurried or pretended. Vital parameters were displayed, intravenous cannulas had to be inserted, medication had to be administered slowly, and they actually had to make the telephone call to request an ambulance. As a consequence, the students felt absorbed in the situation:

We came in to a patient who had a problem and we had to solve it. How do we do this. What do we do, who does what? It was all very concrete. (Focus group 4)

The fact that the scenarios were involving sub-acute medical conditions, taking place in various surroundings (e.g., primary care unit, geriatric ward) and team members arriving one by one as in real life contributed to experiences of being present in a clinical situation.

### Obtaining an outside perspective

Self-reflection was mandatory during debriefings, and the students gave and received feedback from their peers and instructors. The debriefing was appreciated from several points of view; the students were able to gather their thoughts and structure the events that occurred during the scenarios. It was difficult to assess oneself when in the midst of action. Rather, it was easy to focus on mistakes or inaccuracies, and the students were sometimes critical of their own performance, recapitulating mistakes in their minds. To hear what their peers had observed and to receive positive feedback was very supportive in helping the students to gain an outside perspective on their own actions. In line with this, a medical student emphasized:

It was good to hear the others’ perspective on things I thought I didn’t do very well, when they saw it as very good, or other things I had done well, but they found my communication not so clear, and this gave me a feeling what they think is very important, and why it’s important that I do communicate with them. (Focus group 2)

The students expressed that it was essential to reflect on their own actions and to consider what they had done and how they worked together in the team. The students highlighted the fact that during the debriefing, they actually had to talk about what they did well in the scenarios and put words into concrete personal actions. This helped them take the knowledge and experience to a deeper level, although it was odd and unusual to be “forced” to talk about themselves. As one nursing student articulated:

The thing is that you actually articulate the take home message, you say it out loud, what to take with me … you don’t just think it. (Focus group 4)

The observers’ role was important in two ways: observing others and being observed. By observing others, for preparing to give feedback and finding examples, the significance of communication became clear. Feedback from peers was different from feedback from instructors, who the students felt might be too gentle in their criticism. Feedback from other “professions” who had seen them act was significant; the students recognized the other professions’ knowledge in a new and respectful way. Peer students could also note important aspects of knowledge or acting that they observed.

## Discussion

One of the most important findings was that the students emphasized a number of changes in their understanding of teamwork and professional roles after the simulation experience. The students gave vivid details of their impression of being a member of a simulated interprofessional team. They not only displayed a cognitive/theoretical understanding of teamwork fundamentals in accordance with CRM principles but also described the feeling and consequences of putting them successfully into action with the other professions. They revised their views on professional roles and pondered the complexities of communication and teamwork with regard to professional tasks. They described, sometimes with surprise, new discoveries about the other professions, revising perceptions based on experiences from previous clinical rotations. Further, they stressed in positive terms the experience of increasing confidence and esteem for their own and for the other profession. Another important finding was that participation in IPSE offered a rewarding learning experience that differed considerably from other forms of education. They emphasized the importance of hands-on learning compared with mere textbook learning. The benefits of simulation training included being able to make mistakes without embarrassment in front of the other professions and receiving valuable and sincere feedback from instructors and peers, which points to the benefits of positive and appreciative feelings in combination with simulation activities [30]. Moreover, the students described how the experience of stress contributed to a sense of embodied knowledge, emphasizing the significance of the experience of stress as a non-negative issue [31].

With respect to these findings, it is noticeable that the expectation that hierarchies and power relationships in healthcare are obstacles for achieving the learning goals of IPSE might not necessarily be the case [18–20]. While Aase et al. reported on unequal power relations as hindering for learning [18, 19] and nursing students’ hesitation to voice their concerns [20], the present study shows how IPSE can function as a means for promoting collegial communication and mutual trust [16]. The diverging results between prior research of IPSE and the present study indicate that the outcomes of IPSE might be highly sensitive to how the learning environment is designed. Therefore, a critical issue is how IPSE can be arranged to achieve a profound change in students’ perceptions of interprofessional collaboration during the early years of career development [17]. This study and other studies have shown that feeling safe and confident in the learning situation is of central importance [32, 33]. However, there seem to be other design features that are crucial for putting an emphasis on medical aspects (clinical exchange) in comparison with teamwork (collaborative exchange) which might favor medical students [19]. Balancing the two by designing scenario settings where interprofessional collaboration, instead of the individual doctor’s medical knowledge, is crucial for the patient outcome might allow both professions to act on an equal level. Another significant feature could be that the debriefing model used, which was encouraging students to start by describing positive aspects before problematizing what should be improved, could have contributed to a non-threatening and permissive learning environment.

In summary, this study strengthens assumptions on how IPSE should be designed to promote the development of interprofessional competencies. Firstly, it is important to enable an active participation of students from both disciplines, and secondly, it is important to establish a trustful learning environment. One limitation is that we need to be cautious of generalizing the findings to other professional domains. Another restriction is that the findings should not be regarded as to reflect causal relations, for instance, how single design elements affected the outcomes. Establishing the stability of such relations requires a larger data set and hypothesis-driven quantitative studies. It is also important to note that we studied students as “emerging” professionals and so they were still consolidating their own “professional” roles.

## Conclusions

The results point to the potentials of IPSE to thoroughly transform the students’ understanding of the other professions’ roles and responsibilities and present IPSE as a promising means for promoting collegial communication and mutual trust. The students displayed relevant knowledge on fundamental teamwork principles relating to specific simulation activities. A critical condition for developing these new insights was to feel confident in the learning environment, which also contributed to revising prior assumptions on hierarchies and unequal power relations. The significance of the learning environment points to a need for further research considering if and how specific designs can promote role understanding and communicative skills “that truly make a difference to patient care” [2].

## Additional files

Additional file 1: Example of a scenario and the objectives relating to interprofessional collaboration. (DOC 17.1 kb)

Additional file 2: Focus group guide. Focus groups with nursing and medical students. (DOC 18.1 kb)

## Abbreviations

ABCDE: Airway, Breathing, Circulation, Disability, Environment
ATLS: Advanced Trauma Life Support
CRM: Crisis Resource Management
IPSE: Interprofessional simulation-based education
SBAR: Situation, Background, Assessment, Recommendation
