# Correction to: Students’ understanding of teamwork and professional roles after interprofessional simulation—a qualitative analysis

**DOI:** 10.1186/s41077-019-0089-6

**Published:** 2019-02-05

**Authors:** Lena Oxelmark, Torben Nordahl Amorøe, Liisa Carlzon, Hans Rystedt

**Affiliations:** 1 Institute of Health and Care Sciences, The Sahlgrenska Academy, University of Gothenburg, PO Box 457, 405 30 Gothenburg, SE Sweden; 2 Simulation Centre West, Sahlgrenska University Hospital and University of Gothenburg, Gothenburg, Sweden; 3 Department of Education, Communication & Learning, University of Gothenburg, Gothenburg, Sweden

**Correction to: Adv Simul**

**https://doi.org/10.1186/s41077-017-0041-6**

The original article [1] contains an error in the Ethics approval statement of the Declarations regarding approval of the study. The authors would like to note that ethical approval was instead gained by the regional ethics committee in Linköping, Sweden (2012/439-31).
