# Supplementary material for: Students’ understanding of teamwork and professional roles after interprofessional simulation—a qualitative analysis
Source: Adv Simul (Lond). 2017 Apr 8;2:8. doi: 10.1186/s41077-017-0041-6 (PMC5806292; doi:10.1186/s41077-017-0041-6)
Supplement: Supplementary file 1 — Example of a scenario and the objectives relating to interprofessional collaboration. (DOC 17.1 kb) [file 41077_2017_41_MOESM1_ESM.docx]

**Additional file 1:** Example of a scenario and the objectives relating to interprofessional collaboration.

*Summary of a scenario description: It’s a Saturday morning and two nurses are about to go to meet a new patient in the geriatric ward. Eighty-five year old Anna has had a hip-surgery the evening before due to a femur fracture. She has been stable but in need of opiates during the night. The nurses find the patient disorientated, agitated and pale in her bed. She seems to have removed her iv-cannula and urinary catheter. The doctor(s) are not on the ward and needs to be contacted by telephone. (The patient has a hypovolemic chock coming up due to major postoperative bleeding*).

| **Learning objectives:** | **Interprofessional aspects** | **Crucial details in scenario design** |
| --- | --- | --- |
| **ABCDE** | -Who is providing the structure and how is the structure communicated within the team?  -How does the ABCDE structure affect the interprofessional task-sharing?  -How do the different professions contribute to ABCDE structure and patient outcome? | -Questions asked by the debriefer relating specifically to interprofessional aspects  -Needs for the team to discuss different priorities (e.g., stop bleeding, pain relief, fluid therapy, management of agitated patient, replacing urinary catheter)  -Possibility for one profession to start assessment before other profession arrives |
| **SBAR** | -Which expectations are there in the team regarding communication in patient handover?  -How does the team use the different professional competences during handover (“Recommendation”)?  -Are there interprofessional aspects that affect the understanding of a medical situation? | - One of the professions are working on their own in the beginning of the scenario so they need to report the situation to the other profession  -Clinical setting where one of the professions do not have information about the patient and situation  -Parts of the patient history are not relevant to the actual problem (there is a need to sort out important information) |
| **Closed loops** | -How did the different professions contribute to accurate treatment?  -Were there interprofessional misunderstandings and how was this communicated?  -Were the team-members aware of what was done?  -How should the instructions to be given to be clear yet friendly? | -Instructions to the students that every moment of the exercise needs to be done as in reality (e.g., dosages, infusion rate, blood samples)  -The medical condition where the exact dosage and administration of the treatment needs to be discussed and both professions have specific knowledge (e.g., pain relief)  -Team construction and situation where the task sharing is not predetermined (e.g., geriatric ward instead of trauma room) |
| **Speak up** | -Were there different foci in the situation for the different professions? How did the team utilize the different views?  -Were there interprofessional tensions and how were they communicated?  -How did the different professions contribute to patient safety? | -One of the professions having more information from the beginning  -Instructors should not provide clues too early during simulation if the team is struggling (e.g., prompting someone in the team to speak up) |
