# Supplementary material for: Students’ understanding of teamwork and professional roles after interprofessional simulation—a qualitative analysis
Source: Adv Simul (Lond). 2017 Apr 8;2:8. doi: 10.1186/s41077-017-0041-6 (PMC5806292; doi:10.1186/s41077-017-0041-6)
Supplement: Supplementary file 2 — Focus group guide. Focus groups with nursing and medical students. (DOC 18.1 kb) [file 41077_2017_41_MOESM2_ESM.docx]

# Additional file 2

# Focus group guide

# Focus groups with nursing and medical students

*Presenting the aim of the interview*

We would like to hear more about your ideas and thoughts about the possibilities and obstacles using simulation in interprofessional training. In particular, we would like to know more about some of the questions we asked in the course evaluation directly after the simulation. Specifically how you see your own role as nurses/physicians, but also your own role in relation to the physician-/nursing role.

*Organization of the interview*

- Focused discussions
- Distribution of roles
- Around the table questions and responding to others’ contributions

*Opening questions*

- We would like to start by hearing your name and shortly about what impressions still retains from the simulation training (asking each participant)
  - Is there any specific impression/issue you would like to highlight?

*Key questions*

Now we turn to some questions about advantages and disadvantages with simulation and interprofessional learning. Both what simulation may contribute with, but also when simulation is of less relevance.

- What do you think you have learned by simulation training? (could be aspects of collaboration, structured way of working etc)
  - of your own professional role?
  - of others’ professional role?
- Furthermore we would like to hear from each and one of you what it is in/about simulation training that makes learning possible. What was important /of significance specifically for you? (question around)
- Do you find limitations in using simulation training for interprofessional learning?
  - What is not possible to learn from in this way? (eg. related to the future profession?)
  - In what way is simulation hindering in those circumstances?
- Did you find out new issues/things/topics to learn that hadn’t crossed your mind before?
- Was the debriefing of any significance for learning?
  - Questions from the instructors?
  - Feed-back from the instructors?
  - Feedback from other students?
  - The possibility to watch and discuss video recordings?
- Is there any way to improve the prerequisites for interprofessional learning based on simulation? If so, how ?
  - Is there anything in your previous education you find significantly valuable?
  - Is there anything you are missing in your education?
  - Is there anything of specific importance you would like to follow-up after the simulation training?

*Summing up*

- The moderator sums up

*Ending question*

- Is there anything else, such as experiences, impressions or suggestions for improvements that are important in this context that has not been brought up in the interview?

Thank you
